# Gut microbiota-derived metabolite trimethylamine-N-oxide and stroke outcome: a systematic review

**DOI:** 10.3389/fnmol.2023.1165398

**Published:** 2023-06-02

**Authors:** Peng Zhang, Rui Wang, Yang Qu, Zhen-Ni Guo, Yi Yang

**Affiliations:** ^1^Stroke Center, Department of Neurology, First Hospital of Jilin University, Changchun, China; ^2^Neuroscience Research Center, Department of Neurology, First Hospital of Jilin University, Changchun, China; ^3^Department of Epidemiology and Biostatistics, School of Public Health, Jilin University, Changchun, China; ^4^Department of Thoracic Surgery, First Hospital of Jilin University, Changchun, China

**Keywords:** gut microbial metabolism, trimethylamine N-oxide, stroke, prognosis, systematic review

## Abstract

**Introduction:**

The relationship between baseline trimethylamine N-oxide (TMAO) levels and stroke outcomes remains unclear. Therefore, this systematic review aimed to summarize the existing relevant research.

**Methods:**

We searched for studies on the association between baseline plasma levels of TMAO and stroke outcomes in the PubMed, EMBASE, Web of Science, and Scopus databases from their inception to 12 October 2022. Two researchers independently reviewed the studies for inclusion and extracted the relevant data.

**Results:**

Seven studies were included in the qualitative analysis. Among them, six studies reported the outcome of acute ischemic stroke (AIS) and one study of intracerebral hemorrhage (ICH), respectively. Furthermore, no study reported the outcome of subarachnoid hemorrhage. Among patients with AIS, high baseline TMAO levels were associated with unfavorable functional outcomes or mortality at 3 months, as well as a high hazard ratio of mortality, recurrence, or major adverse cardiac event. Moreover, TMAO levels showed predictive utility for unfavorable functional outcomes or mortality at 3 months. Among patients with ICH, high TMAO levels were associated with unfavorable functional outcomes at 3 months, regardless of whether the TMAO value was considered a continuous or a categorical variable.

**Conclusion:**

Limited evidence indicates that high baseline plasma levels of TMAO may be associated with poor stroke outcomes. Further studies are warranted to confirm the relationship between TMAO and stroke outcomes.

## 1. Introduction

In China, ~1.5–2 million newly diagnosed and recurrent cases of stroke occur annually, which makes it the leading cause of acquired disability and mortality among Chinese adults, and thus a huge burden on health resources (Liu et al., 2007, 2011). Therefore, proactive measures for the prompt assessment of risk factors affecting stroke severity and prognosis are required to improve stroke outcomes and reduce the disease burden of stroke.

Gut microbes can influence human health and disease by metabolizing substrates from the diet and host to produce bioactive compounds, including signaling compounds, biological precursors, and toxins (Clemente et al., 2012; Tremaroli and Bäckhed, 2012; Dinan and Cryan, 2017). Trimethylamine N-oxide (TMAO) is an oxidative metabolite produced by gut microbes that metabolize choline-containing lipids and carnitine-like molecules. Circulating TMAO levels are positively correlated with the risk of stroke (Zhang and Yao, 2022). However, the relationship between circulating TMAO levels and stroke outcomes remains unclear. Different studies have explored the relationship between circulating TMAO levels and stroke outcomes with varying stroke subtypes, outcome types, or treatment measures. Therefore, we aimed to conduct a systematic review to summarize the relevant literature.

## 2. Methods

The study protocol of this systematic review was not pre-registered; however, we strictly followed the Preferred Reporting Items for Systematic Reviews and Meta-Analysis (PRISMA) guidelines (Moher et al., 2009).

### 2.1. Search strategy

PubMed, EMBASE, Web of Science, and Scopus databases were searched for studies on the relationship between plasma TMAO levels and stroke outcomes from their inception to 12 October 2022. We used the following search terms: TMAO, stroke, cerebrovascular disease, ischemic infarction, ischemic infarction, ischemic brain infarction, cerebrovascular accident, intracerebral hemorrhage, intracerebral hemorrhage (ICH), subarachnoid hemorrhage, and subarachnoid hemorrhage (SAH). The detailed search strategy used in each database is provided in the Supplementary material (Supplementary Appendix S1). The reference list of each included study was also manually searched for other relevant studies.

### 2.2. Study selection and quality assessment

The eligibility criteria were as follows: (1) original study involving patients with stroke [acute ischemic stroke (AIS), ICH, or SAH]; (2) studies reporting on the relationship between baseline plasma levels of TMAO and stroke outcomes [3-month unfavorable functional outcome (modified Rankin Scale score ≥ 3) or mortality, hazard ratio (HR) of mortality, stroke recurrence, or major adverse cardiac event (MACE)]; (3) studies having included ≥ 100 patients; and (4) those reporting the relevant effect sizes [odds ratio (OR), HR, or area under curve (AUC)], and its corresponding 95% confidence interval (CI). For multiple studies involving the same patient source, the research team determined which study to be included in this systematic review. Retrieved articles were independently evaluated by two authors (P.Z. and Z.N.G.). Differences between the authors were settled through discussions with a third person.

The Newcastle–Ottawa Scale was used to assess the quality of the included studies, which is commonly used for case–control and cohort studies (Wells et al., 2010). It assesses eight items in three major modules: study population selection, comparability, and exposure/outcome evaluation. Two authors (P.Z. and Y.Q.) independently completed the quality evaluation process, with disagreements being resolved through discussion.

### 2.3. Data extraction and analysis

Four authors of the included studies were contacted for additional information. However, no responses were received. We extracted the following information from the included studies: first author, year of publication, country, number of included patients, mean age, sex ratio, treatment, characteristics of the TMAO detection methods, and covariates adjusted in the multivariable model. Two authors completed the data extraction process. First, one author (Y.Q.) independently extracted the data from the included studies, and then, the data were checked by another author (Z.N.G.). We did not perform data synthesis, given the large among-study heterogeneity and the limited number of articles available.

## 3. Results

### 3.1. Search results of the included studies

**Figure 1:**
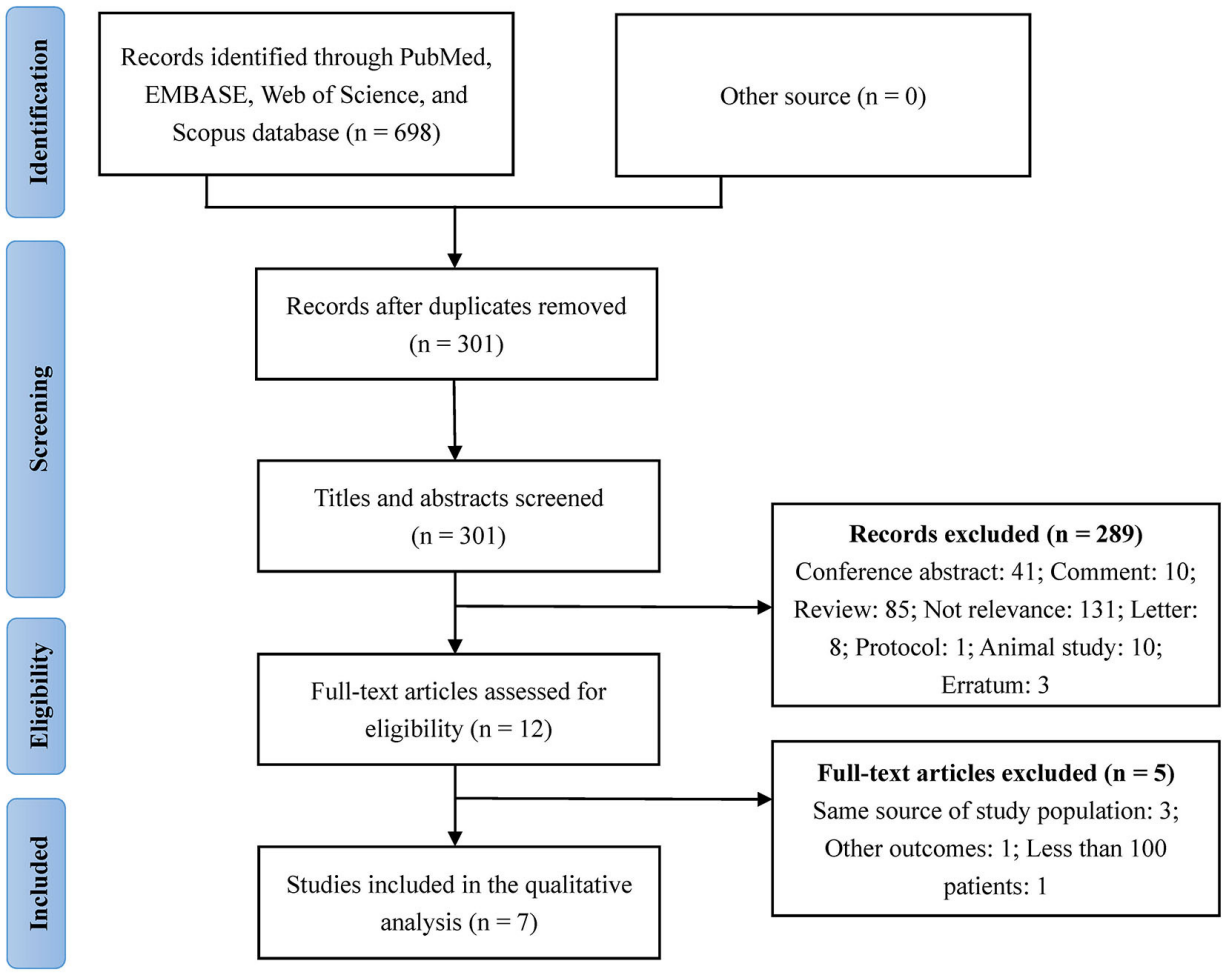
Summary of the study selection process.

We retrieved 698 articles from PubMed, EMBASE, Web of Science, and Scopus databases. There were no relevant articles detected from other sources. After removing duplicate articles, we screened the titles and abstracts of 301 articles; among them, 289 articles were excluded at this stage [conference abstract (*n* = 41), comment (*n* = 10), review (*n* = 85), not relevant (*n* = 131), letter (*n* = 8), protocol (*n* = 1), animal study (*n* = 10), and erratum (*n* = 3)]. Of the remaining 12 articles eligible for full-text screening, 3 articles were excluded for having the same source of the study population, for reporting outcomes other than those relevant to this study (*n* = 1), and for having a sample size <100 (*n* = 1). Finally, seven articles were included in the qualitative analysis. Figure 1 shows the detailed process of article screening. The seven included studies were published between 2019 and 2022; furthermore, six and one studies were conducted in China and Korea, respectively. Additionally, six and one studies reported outcomes of AIS and ICH, respectively, with no study reporting the outcomes of SAH. Table 1 summarizes the main characteristics of the included studies.

**Table 1:** Baseline characteristics of the included studies.

**Study**	**Country**	**Disease**	**Number of patients**	**Mean age, y**	**Male, %**	**Treatment**	**Sample**	**Time from onset to admission**	**Time from admission to sample collection**	**Assay**	**Adjusted for renal function in the multivariable model**
Zhai et al., 2019	China	AIS	225	68.5	55.1	Without EVT and IVT	Plasma	Within 24 h	NR	LC-MS/MS	No
Chen et al., 2022	China	AIS with LAA	291	61.68 ± 7.27	54.6	Not limited	Fasting, Plasma	Within 72 h	Within 24 h	LC-MS/MS	-
Zhang et al., 2021	China	AIS	351	66 (55, 74)	50.4	Not limited	Fasting, Plasma	Within 24 h	Within 24 h	HPLC-MS/MS	Yes
Tan et al., 2020	China	AIS	204	59 (IQR: 21)	66.7	Not limited	Fasting, Plasma	Within 72 h	Within 24 h	LC-MS/MS	Yes
Xu et al., 2021	China	IS or TIA	10027	63 (54, 70)	68.6	Not limited	Fasting, Plasma	Within 7 days	Within 24 h	LC-MS/MS	Yes
Zhai et al., 2021	China	ICH	307	66.8	57.7	Not limited	Fasting, Plasma	Within 6 h	Within 24 h	UPLC-MS/MS	No
Nam, 2019	Korea	AIS with LAA, CE or LI	357	68 (57, 75)	62	Not limited	Fasting, Plasma	NR	Within 24 h	LC-MS/MS	Yes

AIS, acute ischemic stroke; LAA, large artery atherosclerotic; IS, ischemic stroke; ICH, intracerebral hemorrhage; CE, cardioembolism; LI, lacunar infarction; NIHSS, National Institutes of Health Stroke Scale; TMAO, trimethylamine-N-oxide; NR, not report; LC-MS/MS, liquid chromatography-mass spectrometry/mass spectrometry; HPLC-MS/MS, high-performance LC-MS/MS; UPLC-MS/MS, ultra-performance LC-MS/MS; NR, not report; IQR, interquartile range.

### 3.2. Quality assessment of the included studies

The Newcastle–Ottawa scale scores for six and one studies were 8 and 7, respectively (Table 2). The quality of these studies was considered to meet the requirements for inclusion in a systematic review.

**Table 2:** Newcastle–Ottawa quality assessment scale (NOS) for the included studies.

**Study**	**Selection**				**Comparability of cohorts on the basis of the design or analysis**	**Outcome**			**Final score**
	**Representativeness of the exposed cohort**	**Selection of the non-exposed cohort**	**Ascertainment of exposure**	**Demonstration that outcome of interest was not present at start of study**		**Assessment of outcome**	**Was follow-up long enough for outcomes to occur**	**Adequacy of follow up of cohorts**	
Zhai et al., 2019	^*^	^*^	^*^	^*^	^*^	^*^	^*^	^*^	8
Chen et al., 2022	^*^	^*^	^*^	^*^	-	^*^	^*^	^*^	7
Zhang et al., 2021	^*^	^*^	^*^	^*^	^*^	^*^	^*^	^*^	8
Tan et al., 2020	^*^	^*^	^*^	^*^	^*^	^*^	^*^	^*^	8
Xu et al., 2021	^*^	^*^	^*^	^*^	^*^	^*^	^*^	^*^	8
Zhai et al., 2021	^*^	^*^	^*^	^*^	^*^	^*^	^*^	^*^	8
Nam, 2019	^*^	^*^	^*^	^*^	^*^	^*^	^*^	^*^	8

### 3.3. Acute ischemic stroke

#### 3.3.1. Three-month unfavorable functional outcome

Four studies examined the association between TMAO levels and unfavorable functional outcomes at 3 months after AIS; among them, three (Tan et al., 2020; Zhang et al., 2021; Chen et al., 2022) and two (Zhai et al., 2019; Zhang et al., 2021) studies treated TMAO levels as continuous and categorical variables, respectively. Furthermore, three studies reported the AUC of TMAO for predicting 3-month unfavorable functional outcomes (Zhai et al., 2019; Tan et al., 2020; Zhang et al., 2021). Notably, one study used different concentration units for TMAO (pg/ml) compared with the other studies (Chen et al., 2022), another study reported log_2_-transformed TMAO levels (Tan et al., 2020), and another study excluded patients who were treated with intravenous thrombolysis or endovascular therapy (Zhai et al., 2019). These studies are shown in Table 3. All four studies reported a positive association between high TMAO levels and unfavorable functional outcomes at 3 months.

**Table 3:** Summary of the association between TMAO levels (μmol/L) and outcomes of acute ischemic stroke reported by the included studies (effect size with its 95% confidence interval).

**Types of outcome**	**Unadjusted data**			**Adjusted data**		
	**Number of studies**	**Results extracted from the included studies**		**Number of studies**	**Results extracted from the included studies**	
**3-month unfavorable functional outcome (mRS** ≥**3)**						
Continuous	3	Chen et al., 2022	1.10 (0.57–2.12)^a^	2	Zhang et al., 2021	1.21 (1.07–1.35)
		Zhang et al., 2021	1.35 (1.25–1.46)		Tan et al., 2020	1.43 (1.02–2.01)^b^
		Tan et al., 2020	1.44 (1.06–1.97)^b^			
Quartered	2	Zhai et al., 2019	Q2 vs. Q1: 1.56 (0.73–3.33)	2	Zhai et al., 2019	Q2 vs. Q1: 2.01 (0.79–5.11)
			Q3 vs. Q1: 2.78 (1.29–5.98)			Q3 vs. Q1: 2.65 (0.96–7.34)
			Q4 vs. Q1: 3.09 (1.43–6.65)			Q4 vs. Q1: 3.63 (1.34–9.82)
		Zhang et al., 2021	Q2 vs. Q1: 2.61 (1.12–6.12)		Zhang et al., 2021	Q2 vs. Q1: 1.43 (0.78–4.02)
			Q3 vs. Q1: 5.43 (2.41–12.26)			Q3 vs. Q1: 3.02 (1.34–6.12)
			Q4 vs. Q1: 12.93 (5.88–28.42)			Q4 vs. Q1: 5.65 (2.87–13.45)
AUC	3	Zhai et al., 2019	0.63 (0.56–0.70)	0	-	-
		Zhang et al., 2021	0.78 (0.72–0.83)		-	-
		Tan et al., 2020	0.65 (0.54–0.71)^b^		-	-
**3-month mortality**						
Continuous	1	Zhang et al., 2021	1.36 (1.23–1.49)	1	Zhang et al., 2021	1.24 (1.06–1.38)
Quartered	2	Zhai et al., 2019	Q2 vs. Q1: 1.60 (0.53–4.83)	2	Zhai et al., 2019	Q2 vs. Q1: 1.43 (0.34–6.05)
			Q3 vs. Q1: 2.52 (0.88–7.20)			Q3 vs. Q1: 1.89 (0.48–7.39)
			Q4 vs. Q1: 5.64 (2.08–15.30)			Q4 vs. Q1: 4.27 (1.07–17.07)
		Zhang et al., 2021	Q2 vs. Q1: 1.73 (0.40–7.46)		Zhang et al., 2021	Q2 vs. Q1: 0.89 (0.43–3.87)
			Q3 vs. Q1: 4.15 (1.12–15.43)			Q3 vs. Q1: 2.29 (0.83–6.03)
			Q4 vs. Q1: 13.61 (3.95–46.82)			Q4 vs. Q1: 5.84 (3.05–16.12)
AUC	2	Zhai et al., 2019	0.69 (0.60–0.77)	0	-	-
		Zhang et al., 2021	0.80 (0.74–0.87)		-	-
**HR of mortality**						
Continuous	1	Xu et al., 2021	-	1	Xu et al., 2021	1.03 (1.00–1.06)
Quartered	1	Xu et al., 2021	Q2 vs. Q1: 0.81 (0.59–1.12)	1	Xu et al., 2021	Q2 vs. Q1: 0.93 (0.67–1.30)
			Q3 vs. Q1: 0.91 (0.66–1.24)			Q3 vs. Q1: 1.12 (0.80–1.56)
			Q4 vs. Q1: 1.43 (1.08–1.90)			Q4 vs. Q1: 1.39 (1.02–1.90)
**HR of recurrence**						
Quartered	1	Xu et al., 2021	Q2 vs. Q1: 1.10 (0.90–1.35)	1	Xu et al., 2021	Q2 vs. Q1: 1.06 (0.87–1.30)
			Q3 vs. Q1: 1.22 (1.00–1.49)			Q3 vs. Q1: 1.20 (0.98–1.47)
			Q4 vs. Q1: 1.59 (1.32–1.91)			Q4 vs. Q1: 1.54 (1.26–1.88)
**HR of MACE**						
Continuous	1	Xu et al., 2021	-	1	Xu et al., 2021	1.02 (1.01–1.04)
Dichotomous	2	Chen et al., 2022	High vs. low: 4.16 (1.39–12.43)	2	Chen et al., 2022	High vs. low: 3.13 (1.02–9.61)
		Nam, 2019	TMAO cut off: 1.77 (1.11–2.83)		Nam, 2019	TMAO cut off: 1.69 (1.03–2.77)
Quartered	1	Xu et al., 2021	Q2 vs. Q1: 1.06 (0.88–1.27)	1	Xu et al., 2021	Q2 vs. Q1: 1.02 (0.85–1.23)
			Q3 vs. Q1: 1.16 (0.97–1.38)			Q3 vs. Q1: 1.13 (0.94–1.36)
			Q4 vs. Q1: 1.55 (1.31–1.83)			Q4 vs. Q1: 1.45 (1.21–1.74)

^a^pg/ml for baseline TMAO.

^b^log_2_-transformed baseline TMAO.

TMAO, trimethylamine-N-oxide; mRS, modified Rankin Scale; AUC, area under the curve; MACE, major adverse cardiac event.

#### 3.3.2. Three-month mortality

Two studies reported the relationship between TMAO levels and the 3-month mortality after AIS; among them, one (Zhang et al., 2021) and two (Zhai et al., 2019; Zhang et al., 2021) studies treated the TMAO concentration as continuous and categorical variables, respectively. Additionally, two studies reported the AUC of TMAO for predicting 3-month mortality (Zhai et al., 2019; Zhang et al., 2021). One study excluded patients who received intravenous thrombolysis or endovascular therapy (Zhai et al., 2019). Table 3 shows detailed information. Both studies reported a positive association between high TMAO levels and the 3-month mortality.

#### 3.3.3. HR of mortality, stroke recurrence, or MACE

One study examined the relationship between TMAO levels and the HR of mortality (Xu et al., 2021), another study examined the HR of recurrence (Xu et al., 2021), and three other studies examined the HR of MACE (Table 3) (Nam et al., 2019; Xu et al., 2021; Chen et al., 2022). These studies showed that high TMAO levels were associated with shorter survival of mortality, recurrence, and MACE.

### 3.4. Intracerebral hemorrhage

One study explored the association between TMAO levels and outcomes in patients with ICH. Zhai et al. (2021) reported that, after adjusting for potential confounders, the OR of the highest quartile to the lowest quartile of TMAO levels for unfavorable 3-month functional outcomes (mRS ≥ 3) was 3.65 (95% CI, 1.43–9.30; *P* = 0.007) (Zhai et al., 2021). Additionally, the TMAO level as a continuous variable was independently associated with an increased risk of unfavorable 3-month functional outcomes, with an adjusted OR of 1.26 (95% CI, 1.09–1.45; *P* = 0.003).

## 4. Discussion

We systematically searched for studies on the relationship between baseline TMAO levels and stroke outcomes. Although we included a limited number of studies, both unadjusted and adjusted data indicated a relationship between high TMAO levels and poor post-stroke outcomes. High TMAO levels are associated with unfavorable functional outcomes or mortality at 3 months, as well as shorter survival of mortality, recurrence, or MACE.

TMAO production is dependent on the metabolism of dietary choline and carnitine-based molecules by gut microbiota (Craciun and Balskus, 2012). First, gut microbes enzymatically produce trimethylamine (TMA) from the dietary components; subsequently, TMA enters the circulation and is oxidized to TMAO by flavin-containing monooxygenase in the liver (Wang et al., 2011). TMAO is considered a potential mediator in the pathogenesis of stroke and is closely related to the onset of stroke. Sun et al. demonstrated that elevated TMAO levels may portend an increased risk of first stroke after adjusting for important covariates (Sun et al., 2021). Another study on patients who underwent elective coronary angiography reported higher baseline TMAO levels in patients with MACE than in patients without MACE (Tang et al., 2013). When the MACE components were separately analyzed, TMAO levels showed a significant positive correlation with the risk of stroke (Tang et al., 2013). A recent meta-analysis reported that circulating TMAO levels are positively correlated with stroke risk, with stroke patients having higher levels of TMAO compared to non-stroke patients (Zhang and Yao, 2022). However, elevated levels of TMAO are presumed to be associated with stroke outcomes. Animal studies have shown that high levels of TMAO can increase the size of cerebral infarcts and lead to functional deficits, thus directly affecting the severity of stroke (Zhu et al., 2021). Recent clinical studies have also reported a relationship between elevated TMAO levels and poor prognosis in stroke patients (Zhai et al., 2019; Zhang et al., 2021). In this systematic review, we have systematically summarized the relevant literature, but the evidence remains insufficient, necessitating further research to clarify the relationship between TMAO and stroke outcomes.

TMAO is closely related to renal function. TMAO is excreted by the kidneys; accordingly, patients with poor renal function have increased plasma TMAO levels (Rhee et al., 2013; Tang et al., 2015). Circulating TMAO levels in patients with renal dysfunction are negatively correlated with renal function; moreover, abnormally high TMAO levels gradually recover after kidney transplantation (Stubbs et al., 2016). However, renal function is also associated with stroke outcomes. Studies have indicated that chronic renal failure accelerates atherosclerosis and arterial calcification even though the underlying mechanism remains unclear (Buzello et al., 2003; Massy et al., 2005). This may include increased blood levels of calcium, phosphate, and intact parathyroid hormone, as well as perturbed cholesterol metabolism and increased homocysteine levels (Massy et al., 2005; Spence et al., 2016). Recent meta-analyses have demonstrated a relationship between renal impairment at admission with 3-month poor functional outcomes and mortality in patients with AIS treated with intravenous thrombolysis or endovascular thrombectomy (Malhotra et al., 2020; Wang et al., 2022). To summarize, baseline renal function is an important confounding factor, and assessing baseline renal function is essential to elucidating the relationship between TMAO and stroke outcomes.

One possible explanation for the effect of TMAO on stroke outcome involves the activation of the inflammatory state, which is crucially involved in the development and propagation of stroke (Wang et al., 2007; Siniscalchi et al., 2016). TMAO activates the NLRP3 inflammasome by inducing the expression of inflammatory cytokines and adhesion molecules (Seldin et al., 2016; Boini et al., 2017; Chen et al., 2017; Nam, 2019), which contributes to the disruption of the blood–brain barrier and neuronal regeneration (Yang et al., 2019). Another possible explanation is that TMAO is directly involved in platelet hyperreactivity (Zhu et al., 2016; Nam, 2019). Studies have shown that platelet hyperreactivity has adverse effects on the severity and clinical outcomes of cardiovascular diseases (Angiolillo et al., 2007; Schwammenthal et al., 2008). Thus, high TMAO levels may lead to poor outcomes in patients with stroke by modulating platelet function. At present, the mechanisms underlying the prognostic impact of TMAO on stroke have not been determined, and further research is needed.

This study has several limitations. First, we included a small number of studies, and there was high between-study heterogeneity. Therefore, caution should be applied when interpreting our findings; moreover, further studies are required to explore the relationship between TMAO and stroke outcomes. Second, all the included studies were conducted in East Asia. TMAO production is dependent on the metabolism of dietary nutrients by gut microorganisms. Individuals in different regions have different diets and may have different characteristics of gut microbiota, which may affect the circulating TMAO levels. Therefore, our findings may not be applicable in other regions. Finally, we could not determine whether publication bias affected our results.

## 5. Conclusion

Overall, the limited evidence indicates that high baseline plasma levels of TMAO may be associated with poor stroke outcomes. Furthermore, baseline TMAO levels have a certain predictive effect on unfavorable functional outcomes or mortality at 3 months after stroke. Further studies are warranted to determine the relationship between TMAO and stroke outcomes.

## Data availability statement

The original contributions presented in the study are included in the article/Supplementary material, further inquiries can be directed to the corresponding authors.

## Author contributions

PZ, YQ, and Z-NG performed the literature review. PZ, RW, and YY wrote the manuscript. RW and YY helped with the outline and manuscript modification. All authors contributed to the manuscript and approved the submitted version.

## References

[B1] Angiolillo D. J. Bernardo E. Sabat,é M. Jimenez-Quevedo P. Costa M. A. Palazuelos J. . (2007). Impact of platelet reactivity on cardiovascular outcomes in patients with type 2 diabetes mellitus and coronary artery disease. J. Am. Coll. Cardiol. 50, 1541–1547. 10.1016/j.jacc.2007.05.049 17936152

[B2] Boini K. M. Hussain T. Li P. L. Koka S. (2017). Trimethylamine-N-Oxide instigates NLRP3 inflammasome activation and endothelial dysfunction. Cell. Physiol. Biochem. 44, 152–162. 10.1159/000484623 29130962 PMC5828122

[B3] Buzello M. Törnig J. Faulhaber J. Ehmke H. Ritz E. Amann K. (2003). The apolipoprotein e knockout mouse: a model documenting accelerated atherogenesis in uremia. J. Am. Soc. Nephrol. 14, 311–316. 10.1097/01.ASN.0000045048.71975.FC 12538731

[B4] Chen M. L. Zhu X. H. Ran L. Lang H. D. Yi L. Mi M. T. (2017). Trimethylamine-N-Oxide induces vascular inflammation by activating the NLRP3 inflammasome through the SIRT3-SOD2-mtROS signaling pathway. J. Am. Heart Assoc. 6, e006347. 10.1161/JAHA.117.006347 28871042 PMC5634285

[B5] Chen Y. Y. Ye Z. S. Xia N. G. Xu Y. (2022). TMAO as a novel predictor of major adverse vascular events and recurrence in patients with large artery atherosclerotic ischemic stroke. Clin. Appl. Thromb. Hemost. 28, 10760296221090503. 10.1177/10760296221090503 35345908 PMC8969508

[B6] Clemente J. C. Ursell L. K. Parfrey L. W. Knight R. (2012). The impact of the gut microbiota on human health: an integrative view. Cell 148, 1258–1270. 10.1016/j.cell.2012.01.035 22424233 PMC5050011

[B7] Craciun S. Balskus E. P. (2012). Microbial conversion of choline to trimethylamine requires a glycyl radical enzyme. Proc. Natl. Acad. Sci. USA. 109, 21307–21312. 10.1073/pnas.1215689109 23151509 PMC3535645

[B8] Dinan T. G. Cryan J. F. (2017). Gut instincts: microbiota as a key regulator of brain development, ageing and neurodegeneration. J. Physiol. 595, 489–503. 10.1113/JP273106 27641441 PMC5233671

[B9] Liu L. Wang D. Wong K. S. Wang Y. (2011). Stroke and stroke care in China: huge burden, significant workload, and a national priority. Stroke 42, 3651–3654. 10.1161/STROKEAHA.111.635755 22052510

[B10] Liu M. Wu B. Wang W. Z. Lee L. M. Zhang S. H. Kong L. Z. (2007). Stroke in China: epidemiology, prevention, and management strategies. Lancet Neurol. 6, 456–464. 10.1016/S1474-4422(07)70004-2 17434100

[B11] Malhotra K. Katsanos A. H. Goyal N. Tayal A. Gensicke H. Mitsias P. D. . (2020). Intravenous thrombolysis in patients with chronic kidney disease: a systematic review and meta-analysis. Neurology 95, e121–e130. 10.1212/WNL.0000000000009756 32554767

[B12] Massy Z. A. Ivanovski O. Nguyen-Khoa T. Angulo J. Szumilak D. Mothu N. . (2005). Uremia accelerates both atherosclerosis and arterial calcification in apolipoprotein E knockout mice. J. Am. Soc. Nephrol. 16, 109–116. 10.1681/ASN.2004060495 15563564

[B13] Moher D. Liberati A. Tetzlaff J. Altman D. G. (2009). Preferred reporting items for systematic reviews and meta-analyses: the PRISMA statement. BMJ 339, b2535. 10.1136/bmj.b2535 19622551 PMC2714657

[B14] Nam H. S. (2019). Gut microbiota and ischemic stroke: the role of trimethylamine N-Oxide. J Stroke 21, 151–159. 10.5853/jos.2019.00472 31161760 PMC6549071

[B15] Nam H. S. Ha J. Ji D. Kwon I. Lee H. S. Han M. . (2019). Elevation of the Gut microbiota metabolite trimethylamine N-Oxide predicts stroke outcome. J Stroke 21, 350–352. 10.5853/jos.2019.00850 31590480 PMC6780019

[B16] Rhee E. P. Clish C. B. Ghorbani A. Larson M. G. Elmariah S. Mccabe E. . (2013). A combined epidemiologic and metabolomic approach improves CKD prediction. J. Am. Soc. Nephrol. 24, 1330–1338. 10.1681/ASN.2012101006 23687356 PMC3736702

[B17] Schwammenthal Y. Tsabari R. Shenkman B. Schwartz R. Matetzky S. Lubetsky A. . (2008). Aspirin responsiveness in acute brain ischaemia: association with stroke severity and clinical outcome. Cerebrovasc. Dis. 25, 355–361. 10.1159/000118382 18305387

[B18] Seldin M. M. Meng Y. Qi H. Zhu W. Wang Z. Hazen S. L. . (2016). Trimethylamine N-Oxide promotes vascular inflammation through signaling of mitogen-activated protein kinase and nuclear Factor-κB. J. Am. Heart Assoc. 5, e002767. 10.1161/JAHA.115.002767 26903003 PMC4802459

[B19] Siniscalchi A. Iannacchero R. Anticoli S. Pezzella F. R. De Sarro G. Gallelli L. (2016). Anti-inflammatory strategies in stroke: a potential therapeutic target. Curr. Vasc. Pharmacol. 14, 98–105. 10.2174/1570161113666150923111329 26411421

[B20] Spence J. D. Urquhart B. L. Bang H. (2016). Effect of renal impairment on atherosclerosis: only partially mediated by homocysteine. Nephrol. Dial. Transplant 31, 937–944. 10.1093/ndt/gfv380 26567910 PMC4876968

[B21] Stubbs J. R. House J. A. Ocque A. J. Zhang S. Johnson C. Kimber C. . (2016). Serum trimethylamine-N-Oxide is elevated in CKD and correlates with coronary atherosclerosis burden. J. Am. Soc. Nephrol. 27, 305–313. 10.1681/ASN.2014111063 26229137 PMC4696571

[B22] Sun T. Zhang Y. Yin J. Peng X. Zhou L. Huang S. . (2021). Association of gut microbiota-dependent metabolite trimethylamine N-Oxide with first ischemic stroke. J. Atheroscler. Thromb. 28, 320–328. 10.5551/jat.55962 32641646 PMC8147013

[B23] Tan C. Wang H. Gao X. Xu R. Zeng X. Cui Z. . (2020). Dynamic changes and prognostic value of gut microbiota-dependent trimethylamine-N-Oxide in acute ischemic stroke. Front. Neurol. 11, 29. 10.3389/fneur.2020.00029 32082246 PMC7005238

[B24] Tang W. H. Wang Z. Kennedy D. J. Wu Y. Buffa J. A. Agatisa-Boyle B. Li X. S. Levison B. S. Hazen S. L. (2015). Gut microbiota-dependent trimethylamine N-oxide (TMAO) pathway contributes to both development of renal insufficiency and mortality risk in chronic kidney disease. Circ Res. 116, 448–455. 10.1161/CIRCRESAHA.116.305360 25599331 PMC4312512

[B25] Tang W. H. Wang Z. Levison B. S. Koeth R. A. Britt E. B. Fu X. . (2013). Intestinal microbial metabolism of phosphatidylcholine and cardiovascular risk. N. Engl. J. Med. 368, 1575–1584. 10.1056/NEJMoa1109400 23614584 PMC3701945

[B26] Tremaroli V. Bäckhed F. (2012). Functional interactions between the gut microbiota and host metabolism. Nature 489, 242–249. 10.1038/nature11552 22972297

[B27] Wang Q. Tang X. N. Yenari M. A. (2007). The inflammatory response in stroke. J. Neuroimmunol. 184, 53–68. 10.1016/j.jneuroim.2006.11.014 17188755 PMC1868538

[B28] Wang R. Xie Z. Li B. Zhang P. (2022). Renal impairment and the prognosis of endovascular thrombectomy: a meta-analysis and systematic review. Ther. Adv. Neurol. Disord. 15, 17562864221083620. 10.1177/17562864221083620 35646161 PMC9133867

[B29] Wang Z. Klipfell E. Bennett B. J. Koeth R. Levison B. S. Dugar B. . (2011). Gut flora metabolism of phosphatidylcholine promotes cardiovascular disease. Nature 472, 57–63. 10.1038/nature09922 21475195 PMC3086762

[B30] Wells G. Shea B. O'connell D. Peterson J. Welch V. Losos M. . (2010). The Newcastle-Ottawa Scale (NOS) for Assessing the Quality of Nonrandomised Studies in Meta-Analyses. Ottawa, ON: Ottawa Health Research Institute.

[B31] Xu J. Zhao M. Wang A. Xue J. Cheng S. Cheng A. . (2021). Association between plasma trimethyllysine and prognosis of patients with ischemic stroke. J. Am. Heart Assoc. 10, e020979. 10.1161/JAHA.121.020979 34816729 PMC9075360

[B32] Yang C. Hawkins K. E. Dor,é S. Candelario-Jalil E. (2019). Neuroinflammatory mechanisms of blood-brain barrier damage in ischemic stroke. Am. J. Physiol,. Cell Physiol. 316, C135–c153. 10.1152/ajpcell.00136.2018 30379577 PMC6397344

[B33] Zhai Q. Sun T. Sun C. Yan L. Wang X. Wang Y. . (2021). High plasma levels of trimethylamine N-oxide are associated with poor outcome in intracerebral hemorrhage patients. Neurol. Sci. 42, 1009–1016. 10.1007/s10072-020-04618-9 32705490

[B34] Zhai Q. Wang X. Chen C. Tang Y. Wang Y. Tian J. . (2019). Prognostic value of plasma trimethylamine N-Oxide levels in patients with acute ischemic stroke. Cell. Mol. Neurobiol. 39, 1201–1206. 10.1007/s10571-019-00714-3 31332666 PMC11452218

[B35] Zhang H. Yao G. (2022). Significant correlation between the gut microbiota-derived metabolite trimethylamine-N-oxide and the risk of stroke: evidence based on 23 observational studies. Eur. J. Clin. Nutr. 10.1038/s41430-022-01104-7 35468932

[B36] Zhang J. Wang L. Cai J. Lei A. Liu C. Lin R. . (2021). Gut microbial metabolite TMAO portends prognosis in acute ischemic stroke. J. Neuroimmunol. 354, 577526. 10.1016/j.jneuroim.2021.577526 33647820

[B37] Zhu W. Gregory J. C. Org E. Buffa J. A. Gupta N. Wang Z. . (2016). Gut microbial metabolite TMAO enhances platelet hyperreactivity and thrombosis risk. Cell 165, 111–124. 10.1016/j.cell.2016.02.011 26972052 PMC4862743

[B38] Zhu W. Romano K. A. Li L. Buffa J. A. Sangwan N. Prakash P. . (2021). Gut microbes impact stroke severity via the trimethylamine N-oxide pathway. Cell Host Microbe 29, 1199–1208. 10.1016/j.chom.2021.05.002 34139173 PMC8288076

