# Supplementary material for: Gut microbiota-derived metabolite trimethylamine-N-oxide and stroke outcome: a systematic review
Source: Front Mol Neurosci. 2023 Jun 2;16:1165398. doi: 10.3389/fnmol.2023.1165398 (PMC10272813; doi:10.3389/fnmol.2023.1165398)
Supplement: Supplementary file 1 [file Table_1.DOCX]

**Supplementary Materials**

**Appendix S1. Detailed search strategy**

**1. Pubmed**

((((((((((stroke) OR ("cerebrovascular disease")) OR ("intracerebral haemorrhage")) OR ("intracerebral hemorrhage")) OR ("subarachnoid haemorrhage")) OR ("subarachnoid hemorrhage")) OR ("ischemic infarction")) OR ("ischaemic infarction")) OR ("ischemic brain infarction")) OR ("cerebrovascular accident")) AND ((trimethylamine n-oxide) OR (TMAO))

**2. Embase**

#1 'trimethylamine n-oxide'/exp OR 'trimethylamine n-oxide'

#2 TMAO

#3 stroke

#4 'cerebrovascular disease'

#5 'intracerebral haemorrhage'

#6 'intracerebral hemorrhage'

#7 'subarachnoid haemorrhage'

#8 'subarachnoid hemorrhage'

#9 'ischemic infarction'

#10 'ischaemic infarction'

#11 'ischemic brain infarction'

#12 'cerebrovascular accident'

#13 #1 OR #2

#14 #3 OR #4 OR #5 OR #6 OR #7 OR #8 OR #9 OR #10 OR #11 OR #12

#15 #12 AND #13

**3. Web of Science**

#1 (TS=(trimethylamine N-oxide)) OR TS=(TMAO)

#2 (((((((((TS=(stroke)) OR TS=("cerebrovascular disease")) OR TS=("intracerebral haemorrhage")) OR TS=("intracerebral hemorrhage")) OR TS=("subarachnoid haemorrhage")) OR TS=("subarachnoid hemorrhage")) OR TS=("ischemic infarction")) OR TS=("ischaemic infarction")) OR TS=("ischemic brain infarction")) OR TS=("cerebrovascular accident")

#3 #1 AND #2

**4. Scopus**

( TITLE-ABS ( "stroke" ) OR TITLE-ABS ( "cerebrovascular disease" ) OR TITLE-ABS ( "intracerebral haemorrhage" ) OR TITLE-ABS ( "intracerebral hemorrhage" ) OR TITLE-ABS ( "subarachnoid haemorrhage" ) OR TITLE-ABS ( "subarachnoid hemorrhage" ) OR TITLE-ABS ( "ischemic infarction" ) OR TITLE-ABS ( "ischaemic infarction" ) OR TITLE-ABS ( "ischemic brain infarction" ) OR TITLE-ABS ( "cerebrovascular accident" ) ) AND ( TITLE-ABS ( "trimethylamine n-oxide" ) OR TITLE-ABS ( "tmao" ) )
