# Supplementary figures and images for: Gut microbiota-derived metabolite trimethylamine-N-oxide and stroke outcome: a systematic review
Source: Front Mol Neurosci. 2023 Jun 2;16:1165398. doi: 10.3389/fnmol.2023.1165398 (PMC10272813; doi:10.3389/fnmol.2023.1165398)

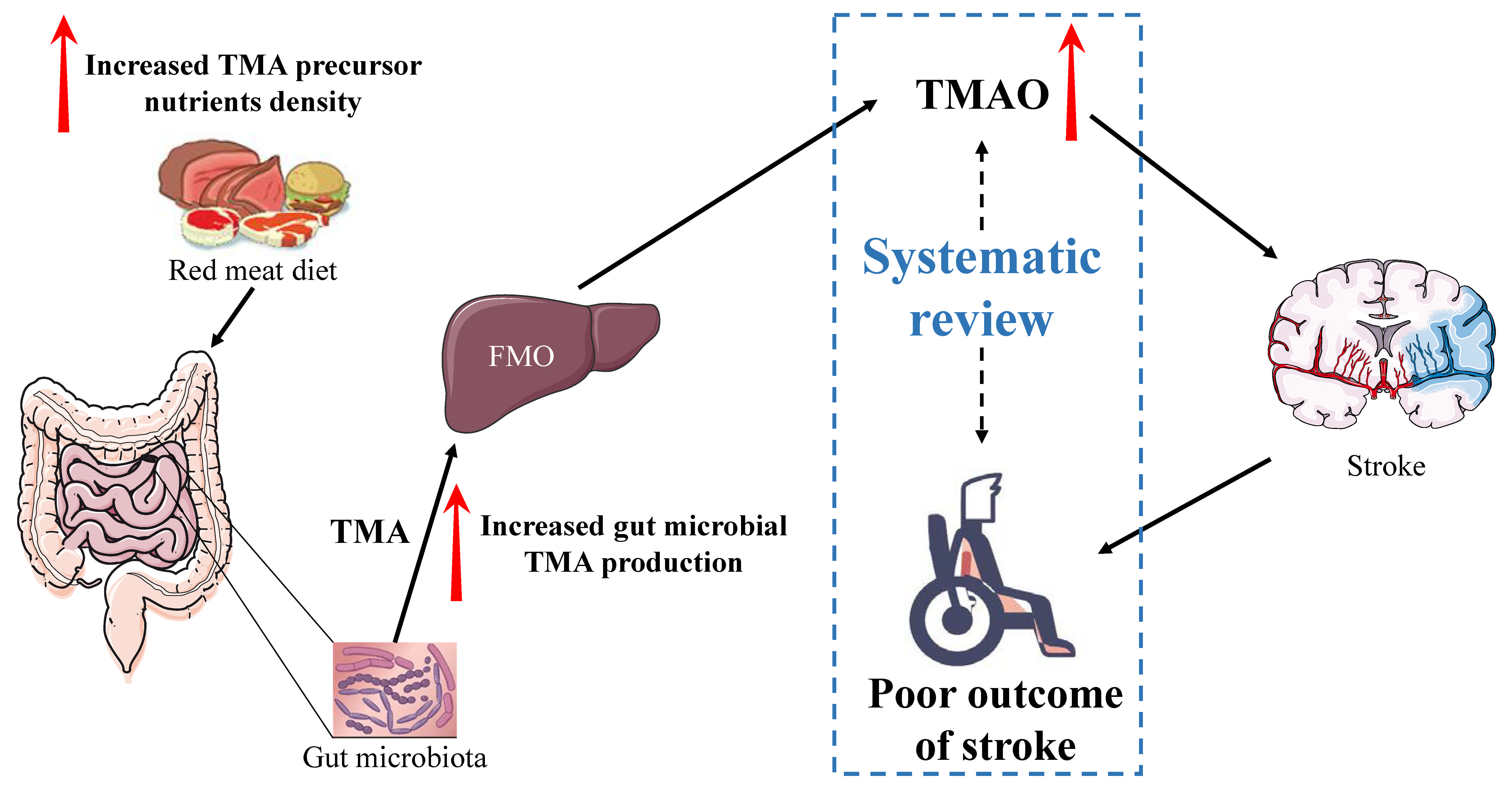

Supplement: Supplementary file 3 [file Image_1.TIFF]
